# Scifer: An R/Bioconductor package for large-scale integration of Sanger sequencing and flow cytometry data of index-sorted single cells

**DOI:** 10.1016/j.immuno.2024.100046

**Published:** 2024-10-29

**Authors:** Rodrigo Arcoverde Cerveira, Klara Lenart, Marcel Martin, Matthew James Hinchcliff, Fredrika Hellgren, Kewei Ye, Juliana Assis Geraldo, Taras Kreslavsky, Sebastian Ols, Karin Loré

**Affiliations:** a Division of Immunology and Respiratory Medicine, Department of Medicine Solna, Karolinska Institutet and Karolinska University Hospital, Center for Molecular Medicine (CMM), Karolinska Institutet, Visionsgatan 18, Stockholm 171 64, Sweden; b Dept of Biochemistry and Biophysics, National Bioinformatics Infrastructure Sweden, Science for Life Laboratory, Stockholm University, Box 1031, Solna SE-17121, Sweden; c Department of Immunotechnology, National Bioinformatics Infrastructure Sweden, Science for Life Laboratory, Lund University, Lund SE-221 00, Sweden

**Keywords:** R package, Bioconductor, Data integration, Sanger sequencing, Flow cytometry, Quality control, Antibodies, B cell receptor, T cell receptor, AIRR

## Abstract

Sanger sequencing remains widely used in various experimental contexts, often in combination with flow cytometry for indexing specific cell populations. However, existing software lacks the capability to automate quality control (QC) of raw Sanger sequencing data and integrate it with flow cytometry information on a large scale. Here, we introduce scifer, an R package now available in the latest release of Bioconductor (3.20) showcasing its effectiveness in seamlessly integrating these types of data as demonstrated by analyses of B cell and T cell receptor sequences. Scifer preprocesses raw data from index sorts and immune receptor Sanger sequencing. It identifies high-quality sequences based on selected parameters, such as length, Phred scores, and heavy-chain complementarity-determining region 3 (HCDR3) quality. As a result, the quality of germline assignments is significantly increased and spurious variable gene mutations are reduced. Scifer is automated and can process thousands of sequences in less than an hour. Its output provides quality control reports, FASTA files, summarized tables, and electropherograms for manual inspection. In summary, scifer is a user-friendly software that speeds up the analysis of immune receptor repertoire sequences, offering wide applicability.

## Graphical Abstract

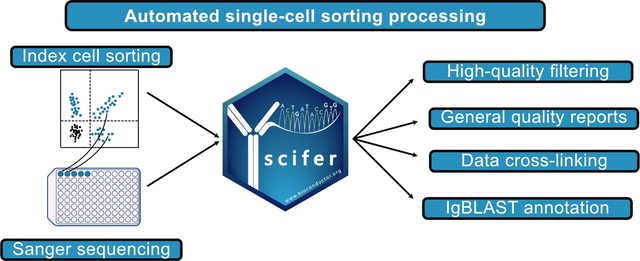

## 1. Introduction

Sanger sequencing has been used for almost 50 years [1]. It became the standard sequencing method to identify germline mutations and validate results for longer amplicons [2]. Concurrently with the development of Sanger sequencing, there were advances in the field of flow cytometry cell sorting [3]. Index cell sorting, a recent mode in flow cytometry, captures fluorescence information during single-cell sorting, enabling retrospective analysis of immunophenotypes from sorted cells [4]. The combined approach is valuable when targeting specific cell subpopulations, leveraging the knowledge of their phenotype during sorting. This method, relying on single-cell index sorting paired with Sanger sequencing, is widely adopted for investigating immune receptor repertoires such as the B cell receptor (BCR) and T cell receptor (TCR).

BCRs are transmembrane-bound immunoglobulins on the surface of B cells and when secreted they are named antibodies [5]. The BCR/antibodies consist of heavy chains and light chains. It is most common to sequence the heavy chain variable genes V, D and J for assessment of nucleotide mutations and quantifying the divergence from germline [6]. There are different workflows for isolating B cells of interest for such analyses. A standard method is based on single-cell flow cytometry sorting of antigen-specific B cells using fluorescently labeled probes of the protein of interest. We and several others have used probes of pathogen proteins when investigating B cells induced by infection or vaccination [7–13]. B cell cultures following cell sorting can be used to screen for antigen-binding antibodies [8,14]. Irrespective of the approach, flow cytometry cell sorting serves as the standard method to identify and isolate B cells of interest preceding sequencing.

Following cell isolation, there are different techniques for sequencing BCRs. These include Sanger sequencing and high-throughput sequencing (HTS) technologies, with HTS being utilized for bulk analysis of targeted amplicon products, as well as for single-cell droplet-based sequencing (scVDJ-seq). Although HTS methods are becoming the standard tool for sequencing, they are less cost-effective than Sanger sequencing for a single amplicon when using a limited number of samples [14]. In addition, Sanger sequencing allows to amplify paired heavy and light chains for downstream antibody cloning, similar to scVDJ-seq. Sanger sequencing is therefore widely used for BCR sequencing, especially when used with lower throughput [14]. Furthermore, when coupled with flow cytometry cell sorting data, simultaneous information of sorted B cells and BCR characteristics can be obtained on both phenotype and antigen-specificity [8]. However, software was lacking that can perform the essential quality control (QC) on raw Sanger sequencing files generated through BCR sequencing and effectively link it with index sort data from flow cytometry cell sorting in an automated manner for large-scale experiments. To address this gap, we developed scifer, an open-source R/Bioconductor package.

Scifer aims to facilitate the preprocessing of raw flow cytometry cell sorting index data and raw Sanger sequencing data. Although scifer was developed focusing on BCR sequences, the filtering process can be customized for any type of sequence and we exemplify this with a TCR dataset. Scifer can also be used solely to process flow cytometry cell sorting index files to extract metadata in an automated manner. The output of scifer includes QC reports, FASTA files, summary tables, and electropherograms for manual inspection.

## 2. Material and methods

### 2.1. Package implementation

**Fig. 1.**
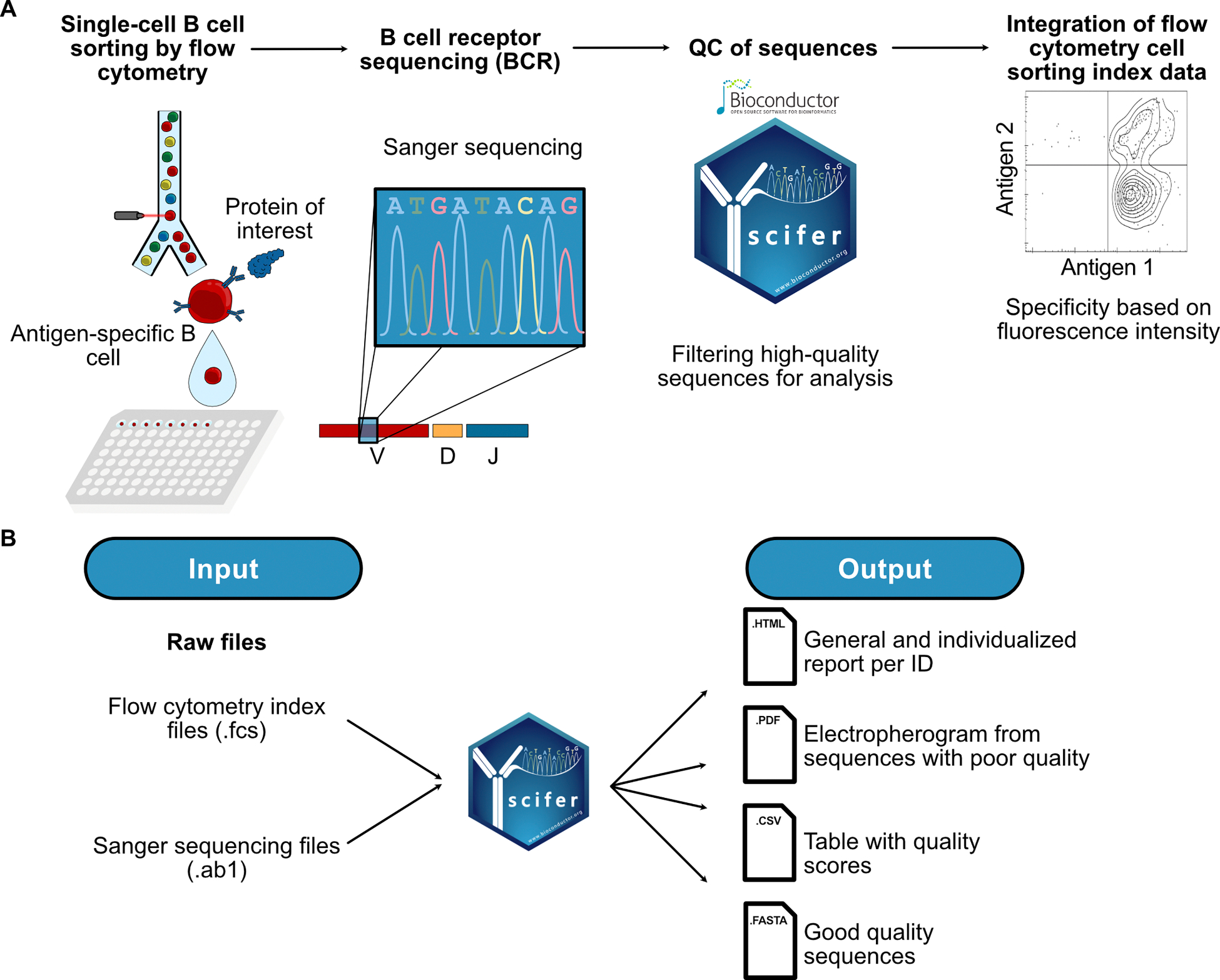
Scifer implementation for processing of Sanger sequences and single-cell flow cytometry cell sorting index data. (A) A typical workflow for single-cell sorting antigen-specific B cells by flow cytometry cell sorting, Sanger sequencing of targeted BCR amplicons, quality control (QC) filtering, and data integration with scifer is depicted. (B) Input data types and all the possible output files generated through scifer.

Scifer is an R/bioconductor package that automates preprocessing of sequences and flow cytometry index sort data. As mentioned, although scifer can be used to process various types of sequences, it was initially developed for BCR sequences which will be exemplified here. The workflow to acquire such data can vary depending on the method implemented in different laboratories. For analysis of B cells, it is common to use fluorescently labeled protein probes as baits to identify the antigen-specific B cells and single-cell sort the cells into 96-well or 384-well plates (Fig. 1A). The plates containing the cells can then be used for targeted PCR amplification of BCRs and Sanger sequencing. After sequencing, raw sequencing files (.ab1) and flow cytometry index data for each cell (.fcs) will be generated. They represent the two input files used by scifer. Neither of them is mandatory, affording the flexibility to utilize one or both inputs based on data availability and the analysis objectives (Fig. 1B). If both datasets are present, scifer will perform a recursive search within the selected folder to match the single-cell index files to the Sanger sequences based on their folder and file nomenclature. The input files of Sanger sequences should be within a folder with a matched name to the flow cytometry data. The first three letters of each Sanger file should be the position of the well as described in detail on the GitHub repository (https://github.com/rodrigarc/scifer). The final output of running scifer includes conventional flow cytometry density plots, a general report for all the sequences, individualized reports per plate, an electropherogram of the approximate position of the complementarity-determining region 3 (CDR3) region with poor quality, a summary table of quality scores, and FASTA files containing the filtered high-quality sequences. Additionally, scifer latest version (v 1.8.0) has an IgBLAST (v 1.22.0) [15] wrapper implemented to facilitate downstream analysis within R software using FASTA files.

### 2.2. Package contents

The package currently contains seven functions that are accessible to the user, as well as a real dataset to test the functions. A standard and practical workflow is to use the *fcs_processing()* function to extract the flow cytometry cell sorting data and plot them using *fcs_plot()* to facilitate defining the thresholds for the fluorescence signals of the antigen probes. After selecting the mean fluorescence intensity (MFI) for what is considered a positive signal of the probe, the parameters are determined for filtering high-quality sequences within the *summarise_quality()* function. The user can customize the filtering parameters or use the default parameters that we have optimized and empirically tested in this study. The *quality_report()* function is a wrapper for most functions to generate and export concise reports, as listed in Fig. 1B. Finally, scifer has an IgBLAST wrapper to align BCR/TCR sequences to databases and returning Adaptive Immune Receptor Repertoire (AIRR) formatted result tables compatible to R objects.

The scifer package includes a small dataset of previously published data [12] in order for users to perform test runs. The entire dataset can be found in the Zenodo repository (8399131). This dataset includes BCR Sanger sequences from index-sorted vaccine antigen-specific B cells obtained from a rhesus macaque. When this dataset was generated, entire single-cell sorted 96-well plates were sequenced including a few wells without sorted B cells, thus offering sequences of poor quality. This creates a good dataset to infer quality thresholds since it consists of many high and low-quality sequences of varying degrees. The immunophenotype of the sorted B cells is characteristic of memory B cells (CD3−, CD14−, CD16−, CD56−, CD19+, CD20+, IgD−, IgG+) of which BCRs bound to Respiratory Syncytial Virus F protein in the prefusion (Pre-F) or postfusion (Post-F) conformation or both. The protocols used to generate this dataset have been described in detail by Ols et al. [12]. There was a clear separation of Pre-F+ and/or Post-F+ B cells, as demonstrated in the package vignette case study (scifer walkthrough vignette).

## 3. Results

### 3.1. Setting default filtering parameters for scifer

**Fig. 2.**
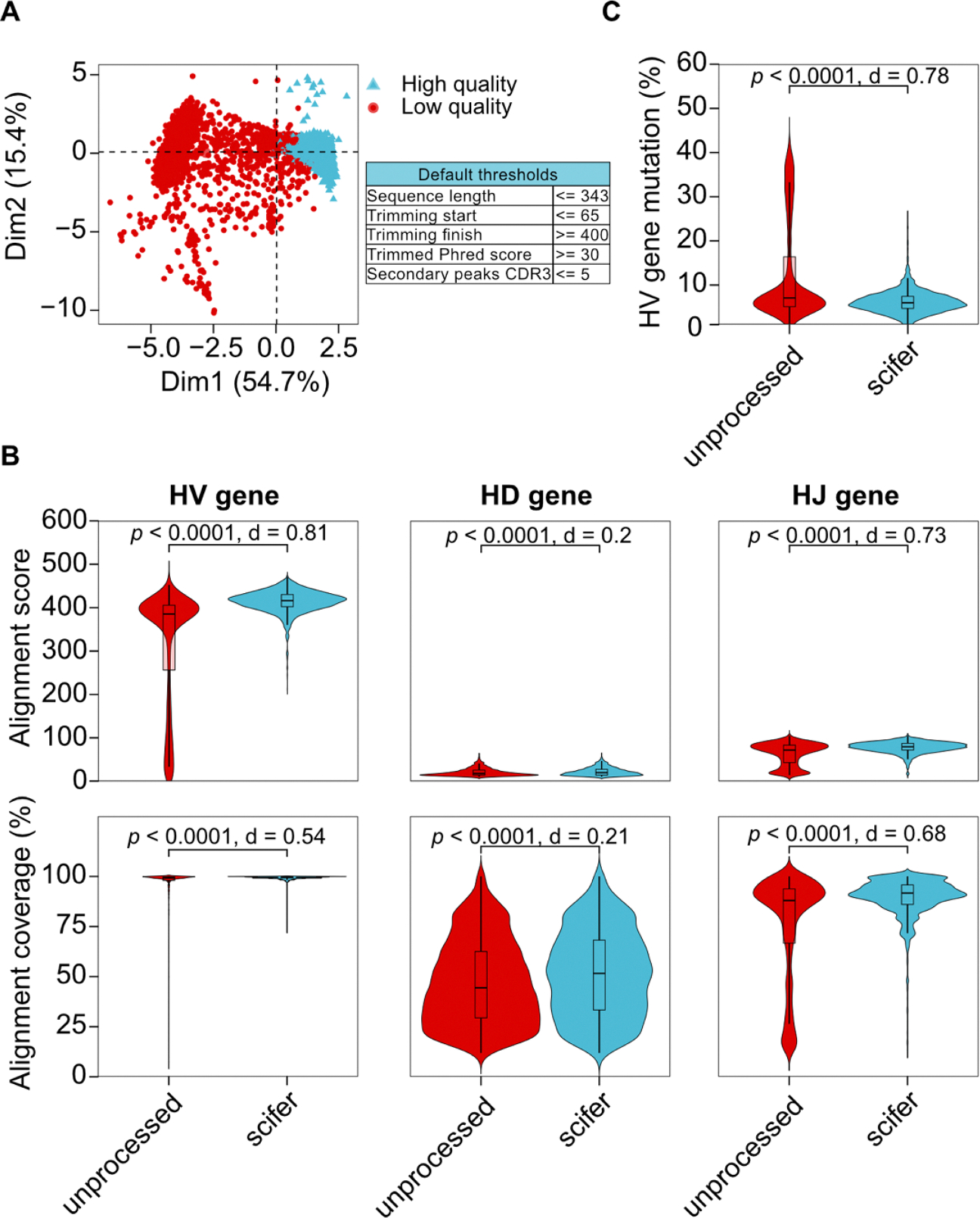
Scifer’s impact on the quality of alignment to BCR genes. (A) Principal component analysis of ten sequence quality variables colored by high quality and low quality according to default thresholds described in the table on the right. *n* = 6244. (B) Comparison of alignment score and alignment percentage coverage to genes between scifer processed and unprocessed BCR sequences. Scifer: Heavy Chain Variable Gene (HV) plots *n* = 3661, Heavy Chain Diversity Gene (HD) plots = 3568, Heavy chain Joining Gene (HJ) plots = 3661; unprocessed: HV plots *n* = 4952, HD plots *n* = 4801, HJ plots *n* = 4851. (C) Heavy chain variable gene (HV) nucleotide mutations between scifer processed and unprocessed sequences. Scifer *n* = 3661, unprocessed *n* = 4952. Student’s *t*-test was used, and the FDR-adjusted p-values are shown on each plot, and Cohen’s d estimates of the effect size.

Default parameters for QC of BCR sequences were established using a dataset of 6244 heavy chain BCR sequences and the corresponding flow cytometry cell sorting data from index-sorted antigen-specific B cells [12]. This data was previously published and includes data from a vaccine study in rhesus macaques (*Macacca mulatta*) where the RSV F protein vaccine antigen was used to identify and sort antigen-binding B cells [12]. To select the filtering criteria, a machine learning approach was used to analyze and separate high and low-quality sequences. Sequence quality parameters were first clustered using the *k-means* algorithm (Supplementary Fig. 1A–B). The independent parameters that explained >5 % of the variance were selected for further analysis (Supplementary Fig. 1C). The selected parameters encompassed six key aspects: raw sequence length, starting and ending positions for good quality base calls (i.e., trimming positions which are used only for filtering but do not trim the output sequences), mean Phred quality score within the good quality positions (i.e., trimmed_mean_quality), and the number of secondary peaks within the approximate CDR3 region set by the user (i.e., cdr3_start and cdr3_end). The filtering threshold for each parameter was set at three standard deviations from the high-quality cluster mean, except for the CDR3 where the threshold was set manually based on the parameter value distribution (Supplementary Fig. 1D–G). A separate threshold for this region was included because the CDR3 is the most variable region and thus comprises a critical section for clonotype analysis. The sequences assigned as high and low-quality (i.e., filtered in or filtered out, respectively) are shown in Fig. 2A. Importantly, these values are set as default but all the filtering parameters can be adjusted by the user based on preference.

### 3.2. Preprocessing with scifer increases overall quality of germline gene alignment

Most analyses of BCR sequencing data require the alignment of query sequences to reference germline gene databases using a tool such as IgBLAST [15]. The effect of preprocessing sequences before alignment was assessed by comparing scifer-processed to unprocessed sequences. Sequences were aligned to a macaque germline allele database, KIMDB [16] using IgBLAST [15] wrapped by IgDiscover [17]. Technical replicates, empty wells, and sequences that had no gene assigned by IgBLAST were removed in both groups, thus decreasing the initial number of 6244 sequences. The quality of the alignment of unprocessed sequences was lower for all genes in the antibody variable region, as shown by the alignment score (Fig. 2B), a measure of the quality and similarity between the query and reference sequences. The alignment coverage, which quantifies the proportion of the query sequence that aligns to the reference, was also significantly lower regardless of the assessed variable region genes. The largest differences were observed for HV and HJ genes (Fig. 2B), which are longer and have lower sequence diversity than the HD genes. The improvement of those metrics was consistent across all HV gene families analyzed (Supplementary Fig. 2A–B). When the alignment score was compared to a single filter of Phred score higher than 30, scifer showed a small but significant quality improvement for the HV gene (Supplementary Fig. 1H). The sequences filtered out by scifer had predominantly a very low alignment score (Supplementary Fig. 1H). Overall, the quality and coverage of alignments significantly increased through scifer preprocessing of BCR Sanger sequences prior to germline assignment.

### 3.3. Filtering for high-quality sequences reduces frequency of spurious mutations

**Fig. 3.**
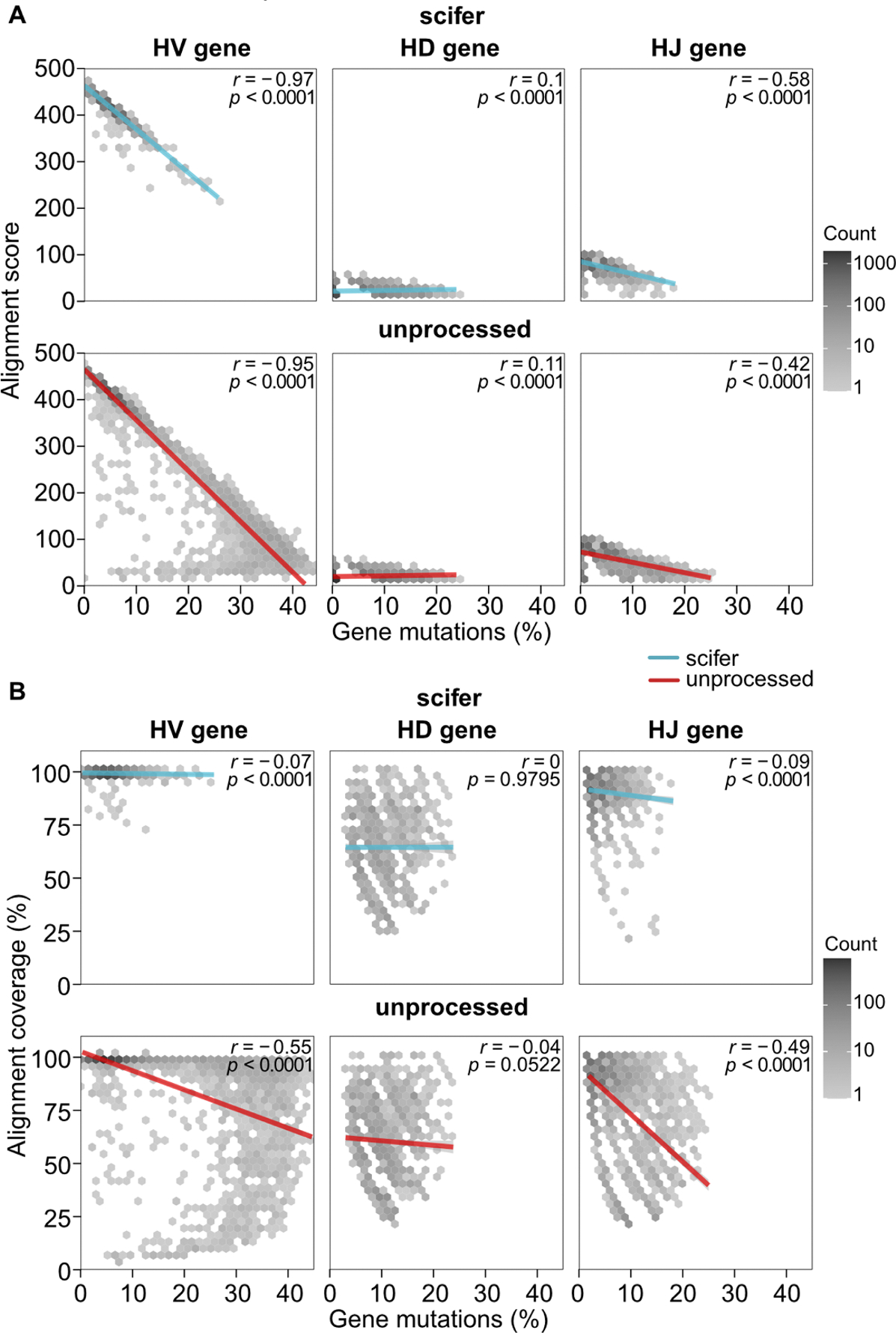
Correlation between heavy chain variable gene nucleotide mutations and germline alignment metrics. (A) Correlation between gene mutations and alignment score. Scifer: HV plots *n* = 3661, HD plots = 3568, HJ plots = 3661; unprocessed: HV plots *n* = 4952, HD plots *n* = 4801, HJ plots *n* = 4851. (B) Correlation between gene mutations and alignment coverage. Scifer: HV plots *n* = 3636, HD plots = 1481, HJ plots = 2396; unprocessed: HV plots *n* = 4922, HD plots *n* = 1875, HJ plots *n* = 3211. Sequences with 0 mutations were removed to reduce bias towards short poorly aligned HD genes. Pearson’s correlation statistical significance and correlation coefficient (*r*) are shown on each plot. Red and blue lines indicate the linear regression between the two variables for scifer-processed or unprocessed BCR sequences.

Following germline alignment, the quantification of mutations acquired is often used as an indication of the affinity maturation status of B cells. However, a low alignment quality might lead to inaccurate identification of mutations. In fact, scifer-processed sequences had a lower percentage of HV gene mutations compared to unprocessed sequences (Fig. 2C and Supplementary Fig. 2C). A strong correlation between the alignment score and the percentage of heavy chain variable (HV) gene mutations was also observed across all HV gene families (Fig. 3 and Supplementary Fig. 2D). Sequences with ~40 % mutations are unlikely to yield productive antibodies. Additionally, poor sequencing quality can overestimate the number of mutations as seen in the strong negative relationship between sequence quality and mutations (Fig. 3A and Supplementary Fig. 3A). When comparing alignment coverage, scifer decreased the negative correlation effect with gene mutations to HV and HJ genes (Fig. 3B). Indicating that mutations identified in scifer-processed sequences are less dependent on sequencing quality. Therefore, removing low-quality sequences prior to alignment provides a better estimate of mutations by reducing spurious mutations inferred due to low sequence quality.

### 3.4. Speed and scalability

Scifer is helpful to avoid manual filtering of BCR sequences, which is usually laborious due to the requirement for manual inspection of electropherograms. The scifer package can process thousands of sequences in less than an hour on a standard laptop (CPU 2.3 GHz Quad-core Intel i7). Scifer’s computations are parallelized, thus it can use multiple cores to speed up the processing and complete a thousand sequences in <30 min (Supplementary Fig. 2B).

### 3.5. Generalizability to other datasets

Although the default settings were set for BCR sequences, they can also be easily adapted to other sequences. A vignette with applicability to TCR sequences is included in the package to facilitate how to identify and change thresholds when necessary. In addition, we have tested the usage of scifer for TCRs from both human and mice datasets, and only minor threshold changes were required (Supplementary Fig. 3A). Thresholds were adjusted according to the expected amplicon length (Supplementary Fig. 3B). The thresholds used for the *Mus musculus* TCR dataset were adjusted to account for a shorter amplicon as a result of the primers used. The length filter was set at 200 and the trimming end position at 250. For detailed information regarding thresholds, please read the package documentation in the README file.

### 3.6. Limitations

At the moment, scifer does not have a built-in batch correction for flow cytometry experiments. Thus, the median fluorescence intensity comparisons can be misleading if there are major batch effects not corrected before using scifer. For this reason, analyzing different batches separately is recommended to decide fluorescence thresholds or using another package to normalize fluorescence across batches. In addition, the default parameters are stringent for high-quality BCR sequences developed based on rhesus macaques, users applying scifer for other sequences and species may require to change the set parameters, in particular their expected amplicon length. Detailed information is included in the package documentation to help the user to change parameters when required. One of these parameters affected by amplicon length is the HCDR3 filter, as precise identification of this region can be complex and differ between BCRs and TCRs, scifer filtering relies on the approximate CDR3 position and length set by the user. Lastly, scifer was first approved for Bioconductor 3.16, thus it has been extensively tested only in R versions greater or equal to 4.2.

## 4. Discussion

A package such as scifer to simplify the task of filtering Sanger sequences for further analysis has long been needed in the BCR repertoire field. Although there are many packages for processing scVDJ-seq and a Python-based package that can cross-link flow cytometry index cell sorting data with BCR sequences exists [18], no R package exists for the processing of raw BCR Sanger sequences and filtering of high-quality sequences. Instead, one can rely on general Sanger sequencing QC not adapted to BCR characteristics [19] and manual integration of flow cytometry index data or perform laborious sequence curation using graphical user interface programs. Scifer was developed to fill this gap by automating the process of filtering high-quality BCR sequences and integrating them with flow cytometry data.

Although scVDJ-seq is becoming the state-of-the-art for BCR sequencing, there are still several issues that need to be addressed. Sanger sequencing is still the most widely used sequencing technique, which also facilitates a broader number of countries to explore the BCR field even without high-cost single-cell sequencing facilities [20]. The cost-benefit for small-scale experiments still favors Sanger sequencing technologies, especially if sequencing needs to be outsourced [14]. Finally, for the discovery of new V and J alleles, Sanger sequencing is still used as the gold standard validation method.

The default parameters set for scifer are applicable for common BCR heavy chain sequences. Although rhesus macaque BCRs were used to set the filtering parameters, the VDJ length is expected to be similar in mice and humans since their HCDR3 regions differ by only a few amino acids [21]. Thus, the length and quality filters should be applicable for at least those species depending on the primer set used. In addition, we have tested scifer for filtering high-quality TCR sequences for both humans and mice. Only the length and final trimming position parameters had to be changed for the mouse dataset due to its shorter amplicons. Furthermore, the user has the option to customize the specific region of interest that is expected to have high quality. In this study, the CDR3 filter was set based on a rough expected position for the CDR3, but the user can tighten or relax the criteria according to their needs. For the aforementioned reasons, and considering that all parameters are customizable, scifer proves highly versatile and applicable to various experimental settings, irrespective of species and primer choices.

Scifer preprocessing increased the average quality of germline alignments for BCR sequences in the datasets tested. Through a comparative analysis of processed and unprocessed sequences aligned to the KIMDB database [16] using IgBLAST [15], scifer-processed sequences consistently exhibited higher alignment scores and coverage across BCR variable region genes, indicating improved sequence quality. Moreover, the study reveals a strong correlation between alignment score and HV gene mutation percentages, underscoring the crucial link between alignment quality and accurate mutation assessment. Although a ground truth for mutation rate is not possible to be calculated, the mean somatic mutation in BCR HV genes in normal memory B cells is between 1 and 3.5 % [16,22,23]. Notably, scifer preprocessing leads to a reduction in spurious mutation estimations by eliminating low-quality sequences. This is particularly important for the immunoglobulin allele discovery field since there are many approaches to infer germlines from sequencing datasets, which are required to be datasets of high quality [24]. These findings highlight the pivotal role of preprocessing and alignment quality in refining mutation analysis and its reliability for BCR research.

Finally, scifer is an Open-Source Software that has undergone code review and approval by Bioconductor. The software is publicly available for download and has already been implemented in multiple studies [11–13].

## 5. Conclusion

We have developed scifer, an R/Bioconductor package, to simplify and accelerate the task of choosing immune receptor repertoire sequences for analysis and expression. It implements data processing from raw flow cytometry index data and Sanger sequencing data developed for BCR sequences but is highly customizable for any type of sequence. It aims to filter the high-quality sequences and integrate them with fluorescence intensity from flow cytometry data. In summary, scifer is a user-friendly software for R users that aims to speed up the analysis of immune receptor repertoire sequences.

## Supplementary Material

supplemental_figures

## Data Availability

Code and data are publicly available on GitHub and Zenodo repositories. The rhesus macaque BCR dataset used is publicly available on Zenodo under 7895251 [12]. Here we have used the raw data from the aforementioned project. The raw data and code used to generate the results are available on Zenodo under 8399141. The scifer package is currently available on Bioconductor 3.20 (https://doi.org/10.18129/B9.bioc.scifer) and on GitHub (https://github.com/rodrigarc/scifer). The analysis presented for this paper is deployed as a GitHub page (https://rodrigarc.github.io/scifer_manuscript/) and the source code is on GitHub (https://github.com/rodrigarc/scifer_manuscript). The mouse and human TCR datasets used to test the package can be provided upon reasonable request.
