# Supplementary material for: Scifer: An R/Bioconductor package for large-scale integration of Sanger sequencing and flow cytometry data of index-sorted single cells
Source: Immunoinformatics (Amst). Author manuscript; Available in PMC 2025 May 5. (PMC12052378; doi:10.1016/j.immuno.2024.100046)
Supplement: supplemental_figures [file NIHMS2067998-supplement-supplemental_figures.pdf]

Supplemental File

Scifer: an R/Bioconductor package for large-scale integration of Sanger sequencing and flow cytometry data of index-sorted single cells

Rodrigo Arcoverde Cerveira[a], Klara Lenart[a,1], Marcel Martin[b,1], Matthew James Hinchcliff[a], Fredrika Hellgren[a], Kewei Ye[a], Juliana Assis Geraldo[c], Taras Kreslavsky[a], Sebastian Ols[a], Karin Loré[a,*]

[a] Division of Immunology and Respiratory Medicine, Department of Medicine Solna, Karolinska Institutet and Karolinska University Hospital; Center for Molecular Medicine (CMM), Karolinska Institutet, Visionsgatan 18, Stockholm 171 64, Sweden

[b] Dept of Biochemistry and Biophysics, National Bioinformatics Infrastructure Sweden, Science for Life Laboratory, Stockholm University, Box 1031, SE-17121 Solna, Sweden

[c] Department of Immunotechnology, National Bioinformatics Infrastructure Sweden, Science for Life Laboratory, Lund University, SE-221 00. Lund, Sweden

[*]Corresponding author:
Karin Loré; karin.lore@ki.se
Division of Immunology and Respiratory Medicine
CMM L8:00
Visionsgatan 18
171 64 Stockholm, Sweden

[1] These authors contributed equally to this work

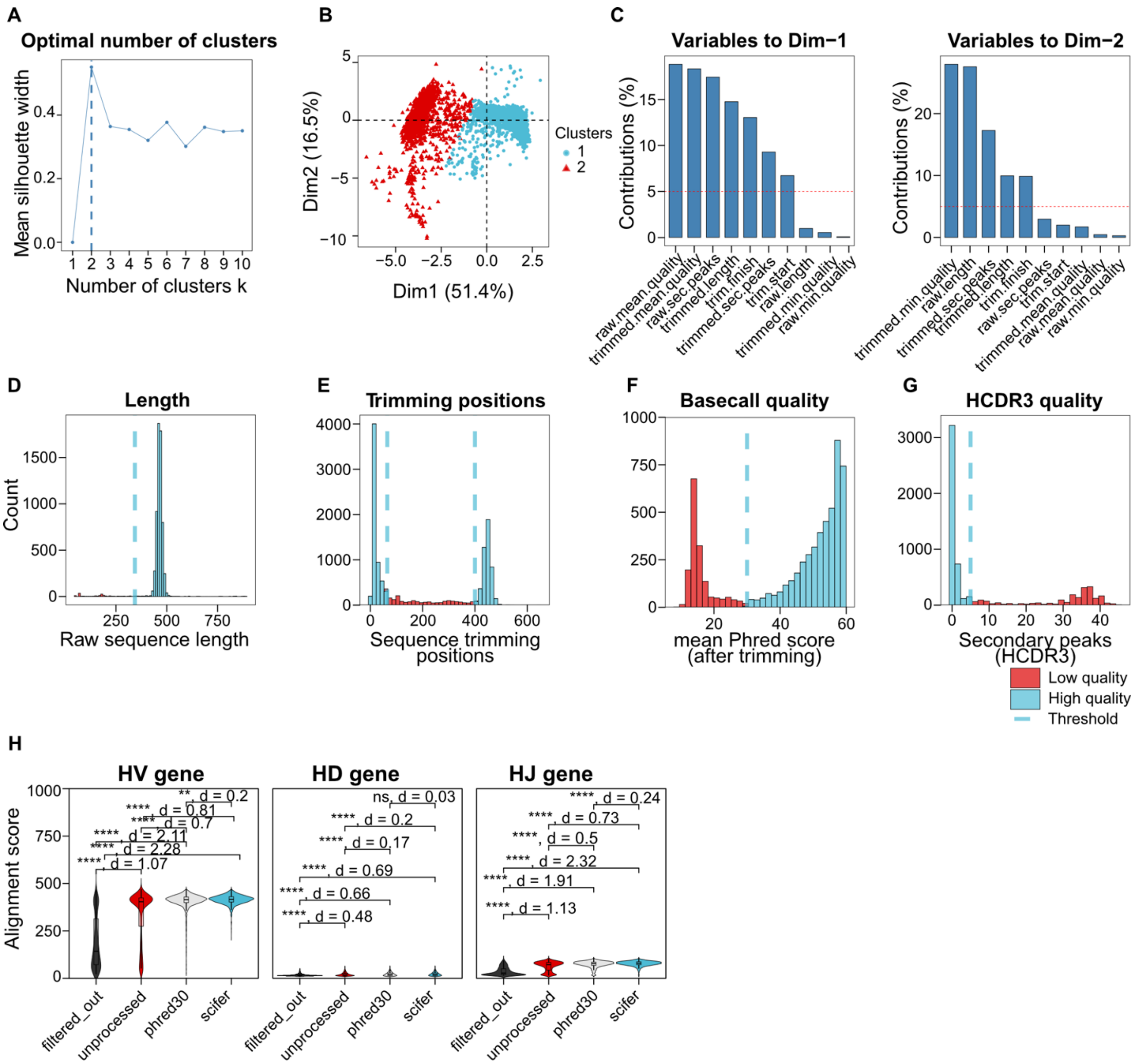

Supplementary Fig. 1 Selection of default filtering parameters for scifer and impact on alignment score. (A) Silhouette algorithm for selection of the optimal number of k-means clusters. (B) Principal component analysis of 10 variables related to quality control from sequences colored by k-means clusters. (C) Variable contributions for the first and second dimensions from the principal component analysis. A horizontal red dashed line marks a 5% contribution to explain the variance. (D-G) Parameters and the threshold used for selection for separating high- and low-quality sequences. The vertical dashed blue line indicates the selected threshold. (H) Alignment score of HV, HD, and HJ genes between scifer filtering with default settings, filtering only with Phred score higher than 30, unprocessed sequences without filtering, and the scifer filtered out sequences. One-way ANOVA test was calculated and Tukey post-hoc test with FDR-adjusted p-values are shown on each plot, Cohen’s d estimates the effect size. ns = not significant, p-value > 0.05; * p-value ≤ 0.05; ** p-value ≤ 0.01; *** p-value ≤ 0.001; **** p-value ≤ 0.0001.

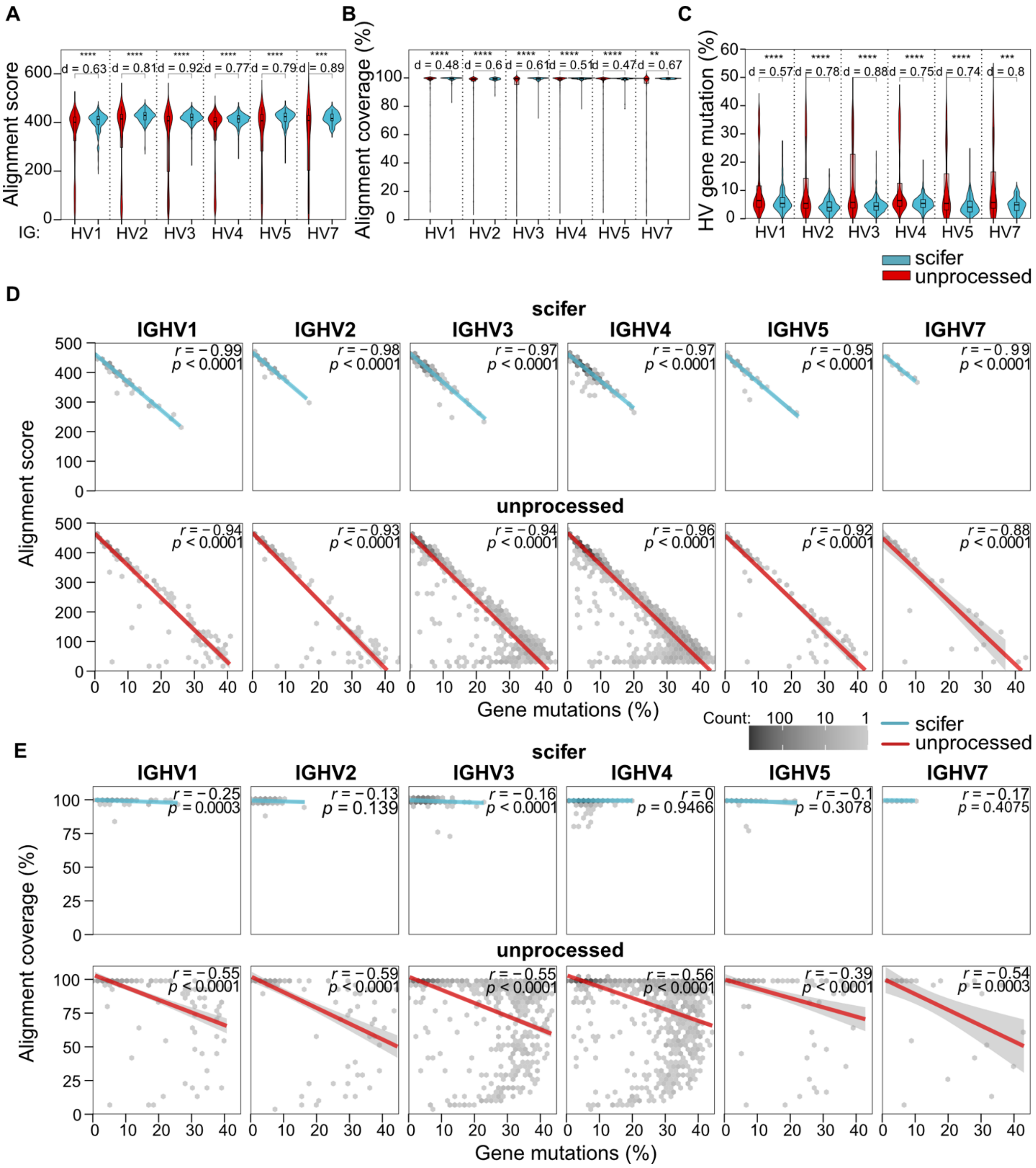

Supplementary Fig. 2 Scifer quality metrics per heavy chain variable gene families. (A) Alignment score between scifer and unprocessed sequences. (B) Alignment coverage between scifer and unprocessed sequences. (C) HV gene mutation between scifer and unprocessed sequences. Student's t-test was used, and the FDR-adjusted p-values are shown on each plot, and Cohen's d estimates of the effect size. (D) Correlation between alignment score and HV gene mutations per gene family. ns = not significant, p-value >

0.05; * p-value ≤ 0.05; ** p-value ≤ 0.01; *** p-value ≤ 0.001; **** p-value ≤ 0.0001. (E) Correlation between alignment coverage and HV gene mutations per gene family. Pearson's correlation statistical significance and correlation coefficient (*r*) are shown on each plot. Red and blue lines indicate the linear regression between the two variables for scifer-processed or unprocessed BCR sequences.

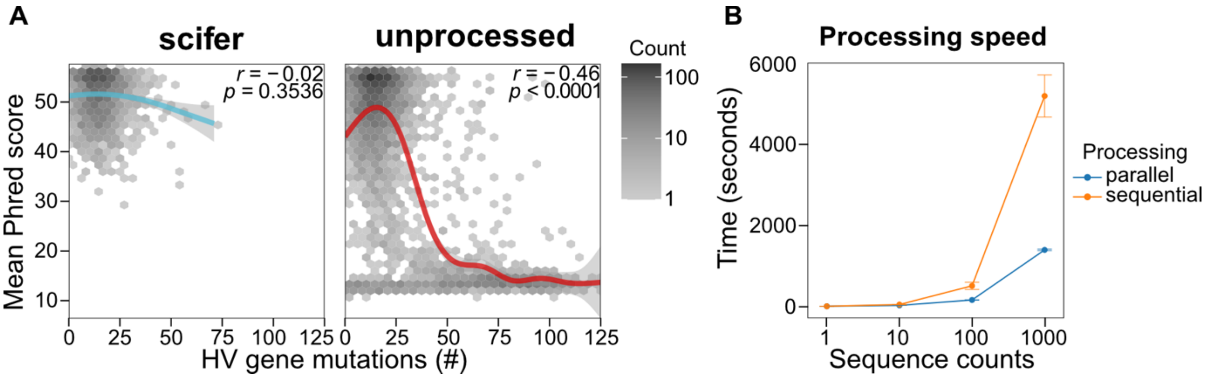

Supplementary Fig. 3 Correlation of mutations with quality and scifer's processing speed. (A) Correlation between the number of HV gene mutations with the mean Phred Score for each sequence. Spearman's rank correlation statistical significance and correlation coefficient (r) are shown on each plot. Red and blue lines indicate the linear model between the two variables for scifer-processed or unprocessed BCR sequences using generalized additive model (GAM). (B) Processing speed on a standard laptop (CPU 2.3 GHz Quad-core Intel Core i7) and demonstration of speed increase due to parallelized processing availability. Error bars represent one standard deviation from two benchmarking replicates.

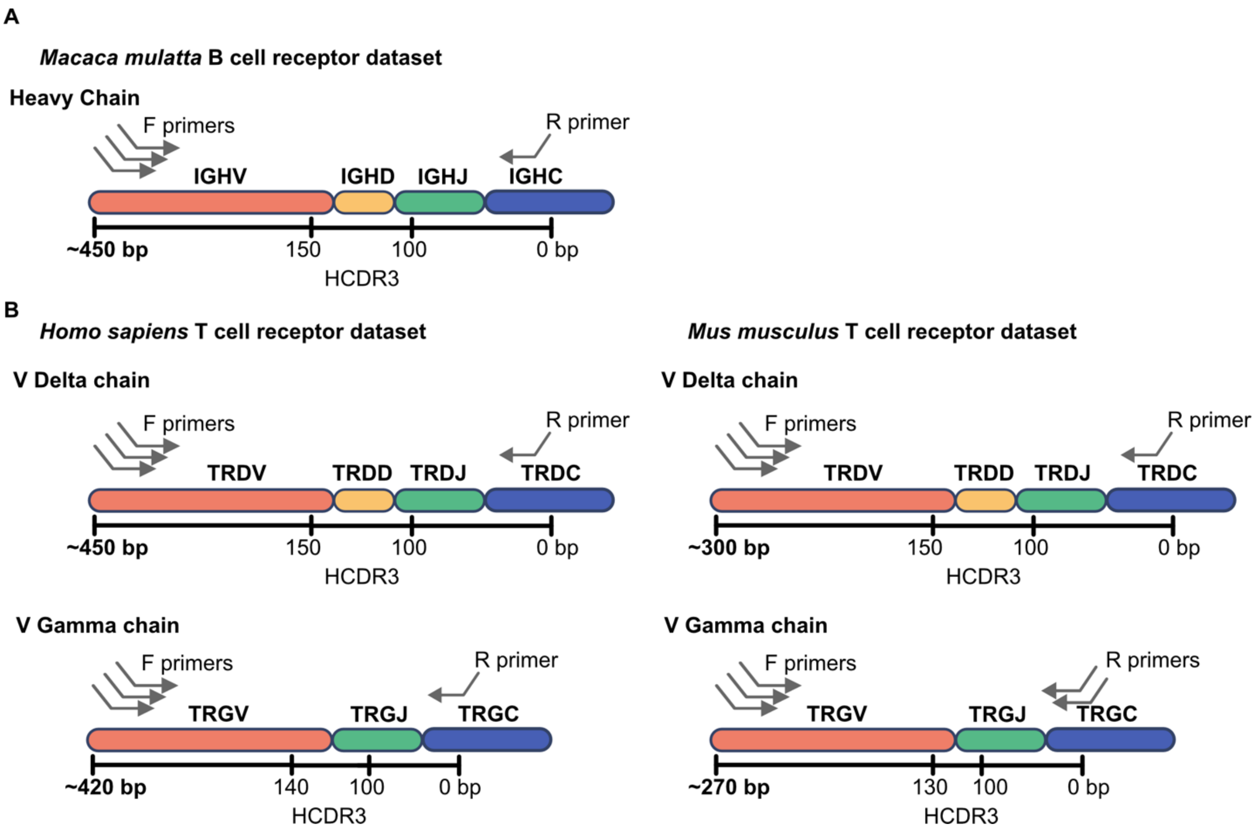

Supplementary Fig. 3 Amplicon differences of VDJ regions from available datasets used for analyses and testing. (A) The main BCR dataset used to set up the thresholds and analyses from rhesus macaques (*Macaca mulatta*). (B) Left: Human (*Homo sapiens*) TCR sequences used for testing the default thresholds for both gamma and delta chain. Right: Mice (*Mus musculus*) TCR sequences used for testing the default thresholds for both gamma and delta chain.
